# Hydroxytyrosol Selectively Affects Non-Enzymatic Glycation in Human Insulin and Protects by AGEs Cytotoxicity

**DOI:** 10.3390/antiox10071127

**Published:** 2021-07-15

**Authors:** Ivana Sirangelo, Margherita Borriello, Maria Liccardo, Marika Scafuro, Paola Russo, Clara Iannuzzi

**Affiliations:** 1 Department of Precision Medicine, Università degli Studi della Campania “Luigi Vanvitelli”, Via L. De Crecchio 7, 80138 Naples, Italy; ivana.sirangelo@unicampania.it (I.S.); margherita.borriello@unicampania.it (M.B.); maria.liccardo@unicampania.it (M.L.); marika.scafuro@unicampania.it (M.S.); prusso@isa.cnr.it (P.R.); 2 Institute of Food Sciences, National Research Council, ISA-CNR, Via Roma 64, 83100 Avellino, Italy

**Keywords:** protein glycation, hydroxytyrosol, antioxidant, AGE toxicity

## Abstract

Hydroxytyrosol (HT), the major phenolic compound in olive oil, is attracting increasing interest for its beneficial properties including a notable antioxidant and anti-inflammatory power. In this study, using a combination of biophysical and cell biology techniques, we have tested the role of HT in the formation of advanced glycation end-products (AGEs). AGEs have a key role in clinical sciences as they have been associated to diabetes, neurodegenerative and cardiovascular diseases. In addition, as the incidence of Alzheimer’s disease (AD) is strongly increased in diabetic patients, AGE formation is supposed to be involved in the development of the pathological hallmarks of AD. Our data show that HT selectively inhibits protein glycation reaction in human insulin, and it is able to counteract the AGE-induced cytotoxicity in human neurotypical cells by acting on SIRT1 level and oxidative stress, as well as on inflammatory response. This study identifies new beneficial properties for HT and suggests it might be a promising molecule in protecting against the AGE-induced toxicity, a key mechanism underlying the development and progression of neurodegenerative disorders.

## 1. Introduction

Neurodegenerative diseases represent different conditions which primarily affect the neurons in the human brain. Currently, neurodegenerative diseases are incurable, and the treatments only affect the symptoms or delay the progression of the disease. Significant research has been devoted to the identification of mechanisms and risk factors involved in the multifactorial etiopathogenesis of these diseases. Recent studies have shown that the accumulation of advanced glycation end-products (AGEs) is a major factor in the incidence and development of several chronic disease, such as diabetes mellitus and its microvascular complications (atherosclerosis, cataracts, and nephropathies), as well as neuropathies [1,2,3,4,5]. In particular, high serum levels of AGEs have been associated to a faster rate of cognitive decline and increasing evidence suggest that AGEs could be implicated in the progression of Alzheimer’s, Parkinson’s disease, and cerebrovascular dementia [4,5,6,7]. In this respect, the incidence of Alzheimer’s disease (AD) is strongly increased in diabetic patients, thus suggesting that AGE formation could be actively involved in the development of the pathological hallmarks of AD [8,9,10,11,12,13]. AGEs are a chemically heterogeneous group of compounds which result from the non-enzymatic glycation reaction between reducing sugars (and their derivatives) and amino-containing biomolecules such as proteins [14,15]. Although the physiological AGE formation is quite slow because of the relatively low reaction temperature, accumulation of AGEs is markedly accelerated by the high glucose levels and oxidative conditions in hyperglycemia and aging [16,17,18,19]. The formation and the accumulation of AGEs can affect protein function through several interrelated mechanisms including protein-protein cross-linking, changes in charge distribution, decreased protein half-life, compromised structural integrity, and altered protein conformation. AGE effect on protein structure depends on different factors including the reactivity of specific amino groups, reactive sugar concentration, and protein half-life [20,21]. In addition, protein glycation has been shown to directly affect the amyloid aggregation process and proteins in amyloid deposits are often found glycated suggesting a direct correlation between protein glycation and amyloidosis [21,22,23,24,25,26].

Besides affecting protein function and structure, AGE species stimulate cells via several receptors including advanced glycation end products (RAGE) inducing oxidative stress, inflammatory response, and gene expression [27,28,29,30]. RAGE signaling and downstream pathways have been implicated in a wide range of inflammatory-related pathologic conditions such as cardiovascular disease, arteriosclerosis, cancer progression, and arthritis [31,32,33,34,35]. The AGE-RAGE signaling has been also implicated in AD pathogenesis and other neurodegenerative diseases [3,36,37]. The AGE-RAGE interaction triggers a series of signal transduction cascades and leads to the activation of transcription factor NF-κB as well as release of pro-inflammatory molecules such as cytokines, chemokines, and adhesion molecules [38,39]. The activation of NF-κB can promote a positive loop that amplify the cellular response to external stress through the upregulation of RAGE expression [40,41]. Moreover, binding of AGEs to RAGE stimulates the generation of reactive oxygen species (ROS) by activation of NADPH oxidase [42,43,44].

Significant efforts have been made to identify compounds able to inhibit the glycation reaction or contrast AGE-induced toxicity with the aim of developing new potential therapeutic strategies [45,46]. In this respect, plant polyphenols have attracted considerable interest due to their multiple biological activities, including anti-oxidant, anti-inflammatory, anti-diabetic, anti-aggregation, and anti-cancer power [47,48,49,50,51]. Mainly, the anti-glycation activity of the phenolic compounds is attributed to the antioxidant activity through the free radical scavenging and dicarbonyl-trapping capacity [50,52].

**Figure 1:**
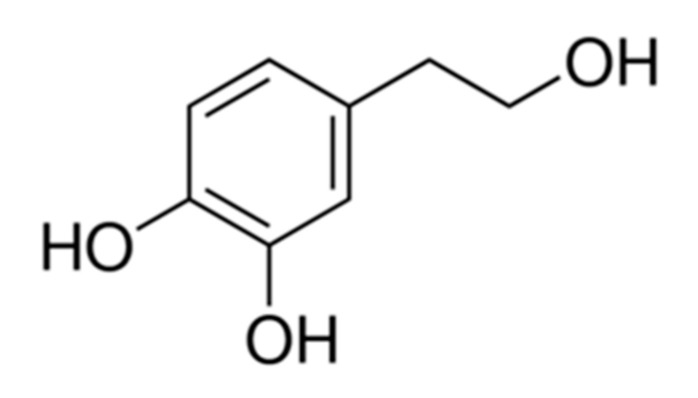
Chemical structure of hydroxytyrosol (HT).

Hydroxytyrosol (3,4-dihydroxyphenylethanol, HT) is the main phenolic compound in olive oil, and it has been widely studied for its beneficial health properties (Figure 1). The free radical scavenging activity and the increase of the endogenous defense systems by HT and its metabolites could be involved in the prevention of different pathologies caused by oxidative stress such as inflammation, cancer, diabetes, cardiovascular, and neurodegenerative diseases [53,54,55,56,57]. Recently, an antiglycation effect exerted by olive leaf extract has also been reported [58].

In this study, we have tested the effect of HT both on glycation reaction and AGE-induced toxicity using human insulin as protein model. Our results show that HT strongly restrains insulin glycation reaction, and it is able to counteract the AGE-induced toxicity by acting on SIRT1 level and oxidative stress, as well as on inflammatory response by inhibiting NF-κB and Erk 1/2 activation.

## 2. Materials and Methods

### 2.1. Materials

Human insulin, hydroxytyrosol, *N*-acetyl-l-tyrosine-ethyl ester, methylglyoxal (MG), d-ribose, 3-(4,5-dimethylthiazol-2-yl)-2,5-diphenyl-tetrazolium bromide (MTT), protease inhibitor cocktail (Sigma-Aldrich Co., St. Louis, MO, USA). Anti-SIRT1 (E104) (ab32441) (Abcam, Cambridge, UK); Anti-SOD2 (D9V9C), Anti-GAPDH (D16H11) (#5174), anti-p42 MAPK (ERK1) (#9102), anti-phospho-p44/42 MAPK (ERK1/2) (Thr202/Tyr204) (#9101) and anti-phospho-NF-κB p65 (Ser536) (#3031) (Cell Signaling Technology, Boston, MA, USA); Anti-α-tubulin (B-7) (sc-5286) (Santa Cruz Biotechnology, Santa Cruz, CA, USA). Secondary antibodies: goat anti-rabbit (GtxRb-003-DHRPX) and goat anti-mouse (GtxMu-003-EHRPX.0.05) (Immunoreagents Inc., Raleigh, NC, USA). SH-SY5Y cell lines (CRL-2266, ATCC, VA, USA). All other chemicals were of analytical grade. MG was further purified by distillation under low pressure and its concentration was determined spectrophotometrically using ε_284_ = 12.3 M^−1^cm^−1^ [59].

### 2.2. Insulin Preparation and Glycation

Human insulin was dissolved in ultra-pure milliQ water to a final concentration of 4 mg/mL at pH 2.0 and protein concentration was determined by absorbance (ε_275_ = 4560 M^−1^cm^−1^). Finally, insulin was neutralized to pH 7.0 and kept in phosphate buffer 50 mM, pH 7.0. HT was dissolved in ultra-pure milliQ water at 100 mM concentration. Glycated insulin was prepared mixing human insulin at a final concentration of 1 mg/mL and 1 mM MG or 0.5 M d-ribose in 50 mM NaH_2_PO_4_ buffer, pH 7.0, passed through a 0.22 μm filter and incubated at 37 °C in sterile conditions in the absence and in the presence of 0.5, 1, and 2 mM HT. Human insulin in the absence and in the presence of HT without glycating agent was used as protein control. Aliquots of protein were collected in sterile conditions and immediately analyzed.

### 2.3. Fluorescence Measurements

Fluorescence measurements were performed on a Perkin Elmer Life Sciences LS 55 spectrofluorometer. To assess the intrinsic fluorescence of AGEs (λex 320 nm/λem 410 nm), glycated insulin at a final concentration of 8 μM was monitored at different incubation times with the glycating agent in the absence and in the presence of HT. The fluorescence intensity was corrected by subtracting the emission intensity of d-ribose/MG solutions at different incubation times. Tyrosine fluorescence emission (λex 275 nm/λem 305 nm) was evaluated on 10 µM glycated insulin after addition of HT at different insulin:HT molar ratio (1:0, 1:0.5, 1:1, 1:2).

### 2.4. Cellular Cultures and Treatments

SH-SY5Y human neuroblastoma cells were cultured in Eagle’s minimum essential medium supplemented with 10% fetal bovine serum, 3.0 mM glutamine, 50 U/mL penicillin and 50 mg/mL streptomycin in a 5.0% CO_2_ humidified environment at 37 °C. Cells were exposed for 24 h to insulin glycated by d-ribose for seven days (final concentration 30 µM) in the absence and in the presence of HT 30 (1:1 molar ratio) and 60 (1:2 molar ratio) µM. The protective effect of HT was monitored at 24 and 48 h by coincubation of cells with 30 µM of fully ribosylated insulin and HT at different molar ratio (1:1, 1:2). For all experiments, untreated cells and cells incubated in the presence of only HT at the tested concentrations served as control.

### 2.5. MTT Assay

Cell viability was assessed as the inhibition of the ability of cells to reduce the metabolic dye 3-[4,5-dimethylthiazol-2-yl]-2,5-diphenyltetrazolium bromide (MTT) to a blue formazan product. After indicated times of incubation with protein samples, cells were rinsed with phosphate buffer solution (PBS). A stock solution of MTT (5 mg/mL in PBS) was diluted ten times in cell medium and incubated with cells for 3 h at 37 °C. After removing the medium, cells were treated with isopropylalcohol, 0.1 M HCl for 20 min. Levels of reduced MTT were assayed by measuring the difference in absorbance between 570 and 690 nm. Data are expressed as average percentage reduction of MTT with respect to the control ± S.D. Data are an average from five independent experiments carried out in triplicate.

### 2.6. Detection of Intracellular ROS

Intracellular ROS were detected by means of an oxidation-sensitive fluorescent probe 2′,7′-dichlorofluorescin diacetate (DCFH-DA). Cells were grown in 12-well plates, pre-incubated with DCFH-DA for 30 min, and then incubated with protein samples for 48 h. Control experiments were performed using untreated cells and cells were exposed to a 0.001 M H_2_O_2_. After incubation, cells were washed twice with PBS buffer and then lysed with Tris-HCl 0.5 M, pH 7.6, and 1% SDS. The non-fluorescent DCFH-DA is converted, by oxidation, to the fluorescent molecule 2′,7′-dichlorofluorescein (DCF). DCF fluorescence intensity was quantified on a Perkin Elmer Life Sciences LS 55 spectrofluorometer using an excitation wavelength of 488 nm and an emission wavelength of 530 nm. Data are expressed as average ± S.D. from five independent experiments carried out in triplicate.

### 2.7. Immunoblotting

Cells were collected by centrifugation, resuspended in lysis buffer (10 mM Tris pH 8.0, 150 mM NaCl, 10 mM NaF, 1 mM dithiothreitol, 1% NP-40, along with the protease inhibitor cocktail and allowed to swell on ice for 20 min. The supernatant was taken after centrifugation at 12,000× *g* at 4 °C for 30 min, and protein concentration was estimated using Bradford’s reagent (BioRad, Hercules, CA, USA). Proteins (25 μg) were separated by 10% SDS-PAGE under reducing conditions and blotted onto a polyvinylidene difluoride membrane in transfer buffer (25 mM Tris, 192 mM glycine, 20% methanol, 0.1% SDS). The blots were then probed with indicated primary antibodies, followed by the corresponding horseradish peroxidase (HRP)-conjugated secondary antibodies. Immunoreactive bands were visualized using an enhanced chemiluminescence detection kit (EuroClone, Milan, Italy) with Chemi Doc XR (Biorad, Hercules, CA, USA). The relative intensity of protein bands was quantified using a Gel Doc XR System (Biorad, Hercules, CA, USA). Densitometry analysis was performed using ImageJ software, processing the digital image and converting the intensity of every protein band in an arithmetic value. Data analysis was performed comparing treated samples vs. control and normalized using the housekeeping gene.

### 2.8. Statistical Analysis

Statistical analyses were performed using Stata software (Version 13.0; StataCorp LP., College Station, TX, USA). Tukey’s post hoc test was used if the treatment was significant on analysis of variance (ANOVA). All data are represented as the mean ± SE. Statistical significance was set at *p* < 0.05.

## 3. Results

### 3.1. HT Effect on Insulin-AGE Formation and Cytotoxicity

**Figure 2:**
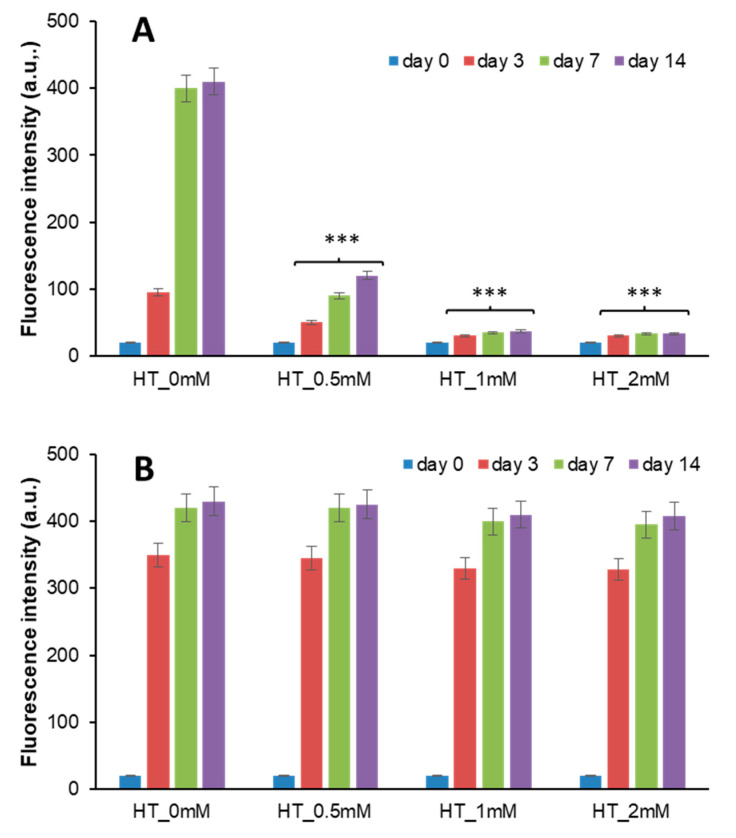
Effect of HT on insulin glycation kinetics. Insulin samples were incubated at 37 °C with 0.5 M d-ribose (**A**) and 1 mM methylglyoxal (**B**) at different concentrations of HT (0–2 mM) and AGE fluorescence (λex 320 nm/λem 410 nm) was monitored at different time points. Other experimental details are described in the Methods section. *** *p* ˂ 0.001 versus sample in the absence of HT (HT_0 mM).

Insulin can be glycated by glucose, d-ribose, and other highly reactive carbonyls, such as methylglyoxal (MG), especially in diabetic conditions [60,61,62,63,64,65,66]. We have previously shown that both d-ribose and MG can react with human insulin producing fully glycated protein in few days [65,67]. To monitor the effect of HT in insulin glycation process, the reaction has been performed both in the presence and in the absence of HT and AGE formation estimated by fluorescence spectroscopy. Indeed, AGEs are characterized by a typical fluorescence emission at 410 nm upon excitation at 320 nm [68]. To this aim, insulin samples have been incubated at 37 °C with 0.5 M d-Ribose or 0.5 mM MG in the presence of different concentrations of HT (0.5; 1; 2 mM), and AGE fluorescence was monitored in time (Figure 2).

As expected, the sample of insulin glycated by d-ribose without HT produced a marked emission intensity at 410 nm during the incubation time and the glycation reaction was completed in about 7 days (Figure 2A). Differently, in the presence of HT, a strong reduction of AGE formation was detected at all incubation times as suggested by a marked decrease of the fluorescence intensity. The inhibition of AGE formation was HT concentration dependent and 1 mM HT was enough to strongly restrain the process (Figure 2A). A different effect was observed for insulin sample glycated by MG (Figure 2B). Indeed, in this reaction the presence of HT did not perturb AGE fluorescence intensity, thus suggesting no effect on insulin-AGE formation.

Interestingly, these data indicate that HT differentially affects AGE formation in human insulin. In particular, while no effect is observed on insulin glycation by MG, a strong inhibitory effect is observed in the presence of d-ribose in a concentration-dependent manner.

**Figure 3:**
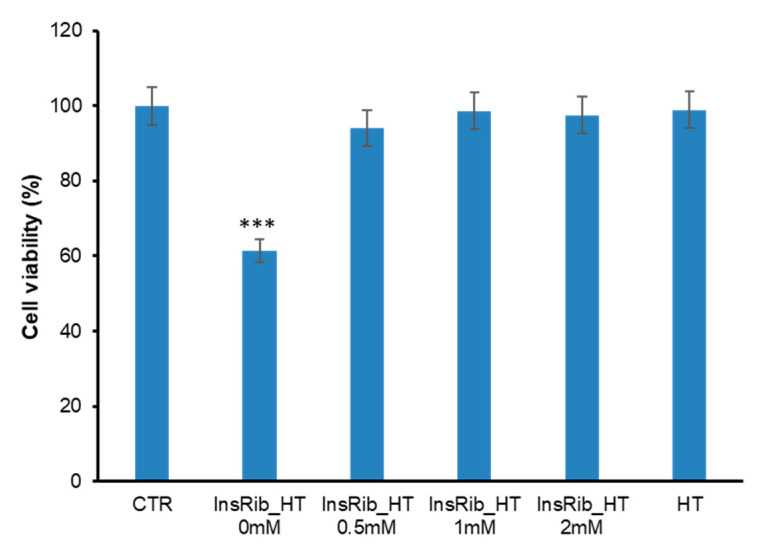
Cytotoxicity of insulin species glycated in the presence of HT. SHSY5Y cells were exposed for 24 h to insulin samples glycated with d-ribose in the presence of different concentrations of HT (0, 0.5, 1, 2 mM) and the cell viability was evaluated by the MTT assay. Data are expressed as average percentage of MTT reduction ±SD relative to control cells from triplicate wells from 5 separate experiments (*p* < 0.01). Other experimental details are described in the Methods section. *** *p* ˂ 0.001 versus CTR.

Further confirmation of HT inhibition on insulin-AGE formation was obtained by cellular toxicity studies. Indeed, it is well established that AGE species promote cell toxicity and we have previously reported that insulin glycation by d-ribose produces AGE species able to strongly affect the cell viability in different cellular models [65,69]. To monitor the cytotoxicity of insulin-AGE formed in the presence of HT, we have monitored the cell viability in neurotypical cells by the MTT assay, which measures the cellular metabolic activity (Figure 3). To this aim, SHSY5Y cells were exposed for 24 h to insulin glycated for 7 days by d-ribose (InsRib) in the absence and in the presence of HT at different concentrations (0; 0.5; 1; 2 mM). As expected, in the absence of HT, fully glycated species reduce cell viability by approximately 40% compared to untreated cells while samples glycated in the presence of HT showed no cell toxicity thus indicating the absence of AGE species in these samples. The same experiment was performed with insulin samples incubated in glycation condition for 14 days and, also in these samples, HT was inhibiting the formation of toxic AGE species (data not shown). These data further indicate that the presence of HT strongly inhibits the formation of AGE species by d-ribose in human insulin.

### 3.2. HT-Insulin Interaction in AGE Formation

**Figure 4:**
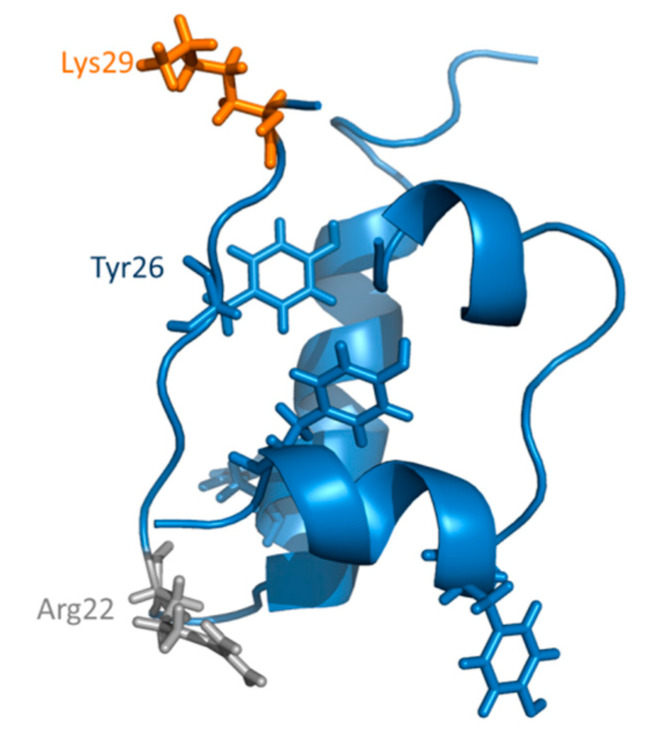
Glycation sites in human insulin. Structural representation of human insulin in its monomeric form (PDB 3AIY). In the tridimensional structure, d-ribose glycation site is represented in orange (Lys29), MG glycation site in gray (Arg22), and tyrosine residues are shown in blue.

**Figure 5:**
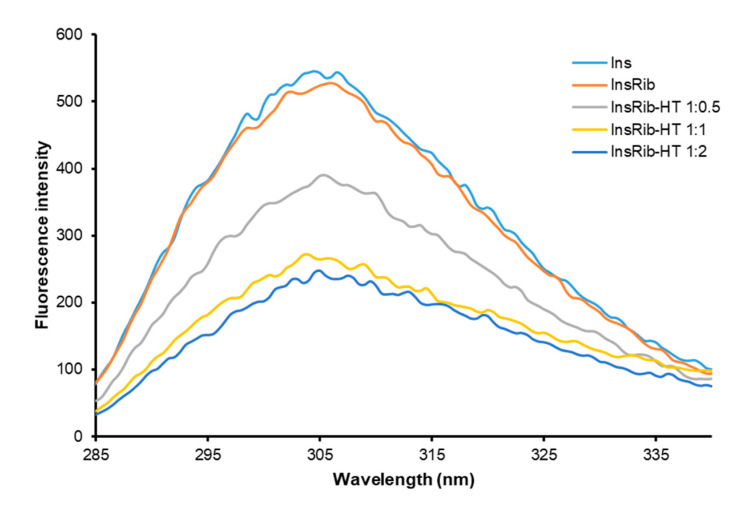
Glycated insulin-HT interaction monitored by intrinsic fluorescence spectroscopy. Tyrosine fluorescence emission has been detected in native insulin (Ins) and insulin glycated by d-ribose (InsRib) in the absence and in the presence of HT at different molar ratio (1:0, 1:0.5, 1:1, 1:2). Insulin working concentration was 10 µM. Other experimental details are described in the Methods section.

We have recently shown that HT specifically binds human insulin in a region close to tyrosine 26 residue in the B chain [70]. As the d-ribose glycation site (LysB29) is located in close proximity to Tyr26 in insulin structure, the binding of HT to human insulin could be responsible for the inhibition of glycation reaction as it could reduce the access of d-ribose to this glycation site (Figure 4). To validate this hypothesis, the glycation reaction has been performed with d-ribose both in the presence and in the absence of HT and the tyrosine emission fluorescence has been monitored at the end of the process (7 days) (Figure 5). Insulin contains four tyrosyl residues as fluorescence emitters and its spectrum is characterized by the typical tyrosyl emission centered at 305 nm. Figure 5 shows the emission fluorescence spectra of insulin glycated by d-ribose (InsRib) in the presence of HT at different molar ratios.

Emission spectra of native insulin in the absence of glycating agent and HT is also shown for comparison. In the absence of HT, the fluorescence emission of glycated insulin almost resembles that of the native protein, thus suggesting no variation in tyrosyl environment upon glycation. Interestingly, the fluorescence intensity regularly decreased in the samples glycated with d-ribose in the presence of increasing concentration of HT, thus suggesting that this molecule induces quenching of tyrosine emission. In addition, as only partial quenching of tyrosyl fluorescence is observed, we can hypothesize that the HT binding only affects few tyrosine residues in insulin structure. The same experiment was performed in the presence of MG as glycating agent but no effect of HT on tyrosyl emission was detected (data not shown), further supporting our hypothesis. The overall data suggest that HT binding might induce small conformational changes in the tyrosyl environment that could selectively reduce accessibility to the close d-ribose glycation site (LysB29).

### 3.3. HT Protective Effect in AGE-Induced Toxicity

**Figure 6:**
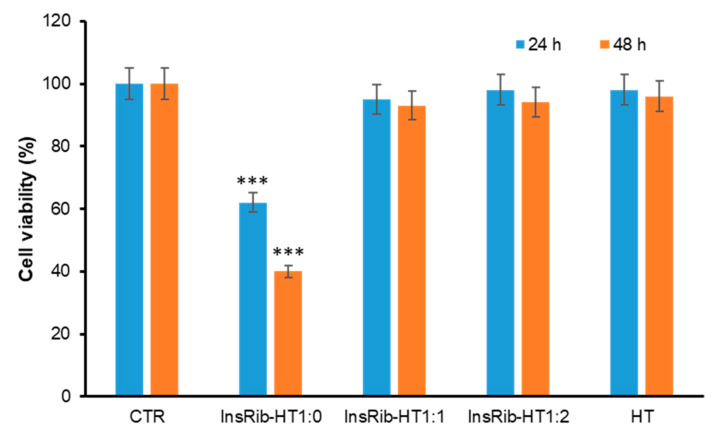
Effect of HT on insulin AGE toxicity. SHSY5Y cells were coincubated with glycated insulin and HT (1:0, 1:1, 1:2 molar ratio) and the cell viability was evaluated after 24 and 48 h by the MTT assay. CTR: cells treated with non-glycated insulin; HT: cells treated with HT at the higher working concentration; data are expressed as average percentage of MTT reduction ±SD relative to control cells from triplicate wells from 5 separate experiments. Other experimental details are described in the Methods section. *** *p* ˂ 0.001 versus CTR.

HT is a phenolic compound proposed to exert a wide range of biological effects, such as cardioprotective, anticancer, neuroprotective, antimicrobial, and anti-inflammatory [54,55,56]. As AGEs are known to induce cytotoxicity through the activation of inflammatory response and oxidative pathways, the ability of HT to protect by AGE toxicity in neurotypical cells exposed to fully glycated species was also investigated as this effect has been never tested before. In particular, the cell viability has been tested by the MTT assay in SHSY5Y cells co-incubated with ribosylated AGE-insulin (InsRib) and HT (1:1 and 1:2 molar ratio) for 24 and 48 h (Figure 6). As expected, glycated insulin induced a strong reduction of the cell viability (40% at 24 h and 60% at 48 h) while, in the presence of HT, no reduction was observed at any time point. Interestingly, HT was able to reverse the toxic effect of insulin-AGEs even at the lower concentration (1:1 molar ratio), thus suggesting a strong protective effect on AGE toxicity.

### 3.4. HT Protective Effect on AGE-Induced Oxidative Stress and Inflammatory Pathways

**Figure 7:**
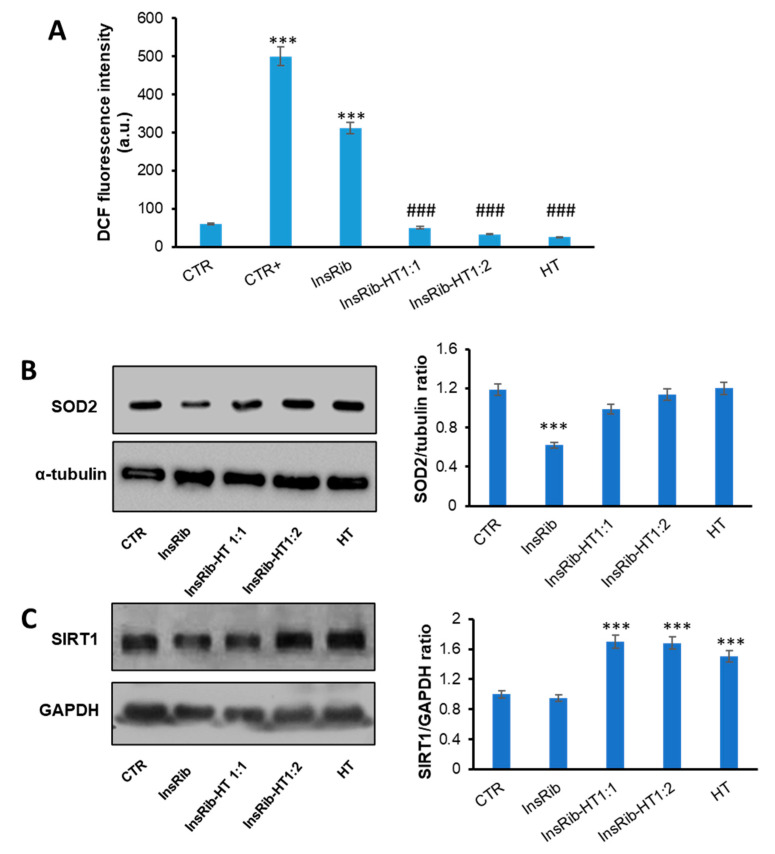
Role of HT in AGE-induced oxidative stress. SHSY5Y cells were co-incubated with glycated insulin (InsRib) and HT (1:0, 1:1, 1:2 molar ratio) and the effect of HT was evaluated in ROS production by DCFH-DA assay (**A**), SOD2 expression (**B**) and IRT1 expression (**C**) by western-blot analysis. CTR: cells treated with non-glycated insulin; HT: cells treated with the higher working concentration of HT; CTR+: cells treated with 1.0 mM H_2_O_2_. Data are expressed as average ± S.D from five independent experiments carried out in triplicate. Other experimental details are described in the Methods section. *** *p* ˂ 0.001 versus CTR, ### *p* ˂ 0.001 versus InsRib.

AGEs are generally responsible, via AGE-RAGE interaction, for an increase of oxidative stress and inflammatory response [29,71]. In order to identify the molecular basis of the cellular protection by which HT counteracts AGE toxicity, we have evaluated the effect of HT both on oxidative stress and inflammatory pathways induced by glycated insulin. At first, we have tested the ability of HT to reduce the AGE-induced ROS production. In particular, as insulin glycated by d-ribose (InsRib) is known to induce ROS production in SHSY5Y cells at 48 h incubation, we have measured the intracellular ROS levels in SHSY5Y cells co-incubated with insulin-AGEs and HT for 48 h, by DCFH-DA fluorescence assay [26,65,72] (Figure 7A). Interestingly, while InsRib promotes ROS production as indicated by the increase in the DCF fluorescence, in the sample co-incubated with HT a strong reduction (more than 85%) of ROS levels was observed, thus suggesting that HT is able to counteract production of oxidative stress. Evaluation of oxidative stress was also performed through the evaluation of SOD2 expression, as mediator of ROS production (Figure 7B). The results show that cells treated with InsRib showed a reduced expression of SOD2 associated to an increase in oxidative stress. Interestingly, SOD2 level in cells cotreated with both InsRib and HT was similar to that of untreated cells, thus indicating that the presence of HT is able to counteract the AGE-dependent oxidative stress and protect cells from AGE-related toxicity.

With the aim of further characterize the protective effect observed for HT in AGE-related oxidative damage, we have also evaluated the role of HT in molecular pathways involved in AGE oxidative stress. In this respect, SIRT1, a protein deacetylase, represents a very important key regulator in cellular protection as its activation markedly protects cells from oxidative stress injury and also hinders inflammatory pathways by inhibition of Nf-κB [73,74]. To probe the effect of HT on SIRT1 levels in AGE-induced toxicity, we have monitored SIRT1 expression in cells treated with InsRib in the presence and in the absence of HT at different concentrations. In particular, cells were exposed for 24 h to glycated insulin at different concentration of HT (1:0, 1:1, 1:2 molar ratio), and SIRT1 levels were evaluated by western-blot analysis (Figure 7B).

Interestingly, while cells treated with glycated insulin showed no variation in SIRT1 expression compared to untreated cells, the ones cotreated with HT (both 1:1 and 1:2 molar ratio) showed an increased expression (80%) of SIRT1. These results suggest that the protective effect observed for HT on AGE-induced oxidative stress is likely associated with the ability of HT to upregulate SIRT1 expression, thus contributing to boosting the cellular antioxidant pathways.

AGEs are also responsible, via AGE-RAGE interaction, for an increase of inflammatory response through the activation of NF-κB, which in turn is responsible for an increased expression of proinflammatory cytokines and for the activation of the MAPK signalling pathway through the phosphorylation of extracellular signal-regulated kinases (ERK1/2) [27,28,71,75,76].

**Figure 8:**
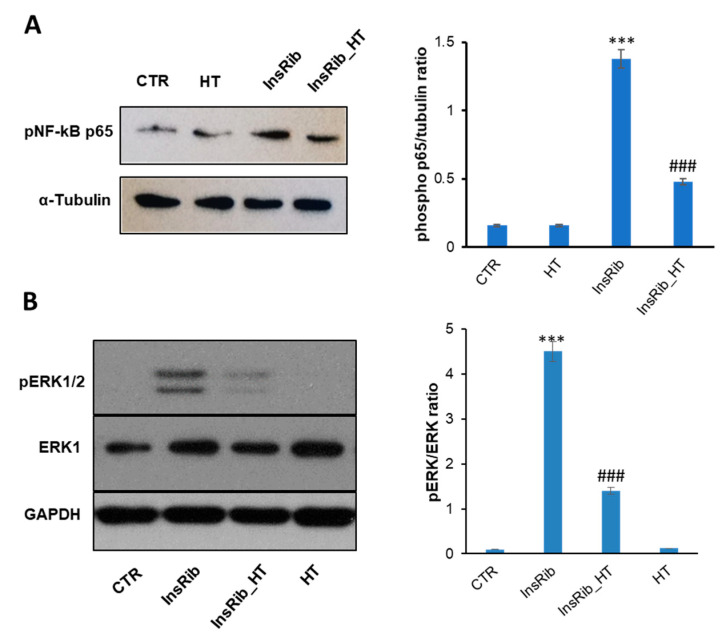
Effect of HT on NF-kB and Erk 1/2 activation. Western-blot analysis of phosphorylated p65 NF-κB (**A**) and Erk 1/2 expression and phosphorylation (**B**) in SHSY5Y cells treated for 24 h with glycated insulin in the absence (InsRib) and in the presence of HT 1:1 molar ratio (InsRib_HT). CTR: untreated cells. HT: cells treated with only HT for 24 h. Working concentrations were 40 µM both for insulin and HT. Other experimental conditions are described in the Methods section. *** *p* ˂ 0.001 versus CTR, ### *p* ˂ 0.001 versus InsRib.

We have recently shown that glycated insulin promotes NF-κB and caspase 3/7 activation in endothelial cells through the activation of the AGE-RAGE signaling pathway [65,69]. To better analyze the protective effect observed for HT in the AGE-related toxicity, we have evaluated both the NF-κB and ERK 1/2 activation in cells exposed to InsRib in the absence and in the presence of HT. To this aim, SHSY5Y cells were co-incubated for 24 h with InsRib and HT (1:1 molar ratio) and activation of NF-κB as well as ERK 1/2 was evaluated by western-blot analysis (Figure 8). As expected, glycated insulin promotes the activation of NF-κB as indicated by a significant increase (8.6 fold) of phosho-NF-κB level compared to untreated cells. Differently, the cotreatment with HT reduces of almost 3-fold the NF-κB activation induced by AGE species, thus suggesting clear anti-inflammation activity (Figure 8A). Similarly, InsRib also promotes ERK 1/2 activation as suggested by a 45-fold increase of enzyme phosphorylation at 24 h incubation compared to untreated cells. Additionally, in this case, the cells co-incubated with glycated insulin and HT showed a reduced enzyme activation (3-fold reduction), thus suggesting that the protective effect observed for HT on AGE toxicity could be due to a modulation of NF-κB/ERK 1/2 pathway. In this respect, the modulation of SIRT1 by HT can both regulate inflammatory response through inhibition of NF-κB activation, and also boost the cellular response to oxidative stress [73,74].

## 4. Discussion

Advanced glycation end-products (AGEs) are very reactive metabolites produced by the nonenzymatic glycation reaction between sugars and biological macromolecules such as protein, DNA, and lipids. AGEs accumulate both intracellularly and extracellularly and contribute both to the onset of multiple diseases and the subsequent disease complications. In fact, AGEs are considered pathological hallmarks in aging and various age-related chronic pathologies such as inflammation, neurodegenerative diseases, atherosclerosis, and vascular complications of diabetes mellitus [1,2,3,4]. In particular, it is well known that AGE accumulation and oxidative stress play a central role in the pathogenesis of neurodegenerative diseases [6,7]. The brain is particularly prone to oxidative damage due to poor antioxidant defenses [77]. AGE accumulation has been observed in brains affected by AD and PD as well as other neurodegenerative disorders [6]. Accumulation of AGE species is associated with increased protein dysfunction and aberrant activation of cellular signaling cascades. Formation of AGE cross-link accumulates over time, and it is stimulated by high glucose levels, as in diabetic patients. In addition, glycation of proteins has been reported to destabilize the native state and stimulate protein aggregation as well as amyloid deposition [21,22,23,24,25,26].

For this reason, much attention has been devoted to the identification of molecular targets able to prevent or limit the glycation reaction and the AGE-dependent cell damage. Several strategies have been developed to prevent the detrimental effect of AGEs, including the employment of natural or synthetic AGE inhibitors and compounds able to interfere with inflammation and oxidative stress. Although several synthetic compounds can efficiently inhibit AGE formation or break protein cross-links, they can be associated with severe side effects. Recently, great attention has been addressed to natural compounds such as polyphenols, well known for their antioxidants and anti-inflammatory properties [54,55,56]. In this respect, HT it has been reported to inhibit the glycation reaction by MG in bovine serum albumin and this effect seems to be ascribed to the ability of HT to specifically trap the MG [78,79]. Indeed, the inhibitory effect was observed only when the protein was glycated in the presence of similar concentrations of HT and MG (molar ratio MG:HT 1:1 and 2:1), while no effect was observed in the presence of MG excess (MG:HT ratio 10:1). In our study we have tested the ability of HT to affect the non-enzymatic glycation of human insulin in the presence of two different glycating agents, d-ribose and MG, used in a large excess to be independent from the potential trapping effect. Insulin is susceptible to in vivo glycation by d-ribose and reactive carbonyls, as MG, especially in diabetic conditions and AGE species are considered the main responsible for diabetes-related vascular complications [17,62,64,80]. Insulin can also be glycated in vitro and, in our conditions, the glycation reaction by d-ribose or MG is completed in few days [49,65]. Our results indicate that HT differentially affects insulin glycation in the presence of d-ribose and MG. Indeed, the presence of HT was hindering the AGE formation by d-ribose, but no effect was recorded in the presence of MG. In insulin glycation, d-ribose is known to react with N-terminus and Lys29 of insulin B-chain while MG with a single site, i.e., Arg22 of insulin B chain [63]. The inhibitory effect observed for HT only in the presence of d-ribose might suggest that HT specifically interacts with insulin in a site close to the Lys29 thus hindering the glycation reaction. Interestingly, Lys 29 is very close to the Tyr26 and the tyrosine quenching induced by the presence of HT could further support this hypothesis. The region between Tyr26 and Lys29 is an unstructured region in human insulin that is likely to be stabilized by HT binding (Figure 4) as also suggested by a recent study in which we have shown that HT specifically binds human insulin in a region close to Lys29 in the B-chain [70]. Our study has also shown that the HT binding prevents the amyloid formation in human insulin by inducing stabilization of the region close to Lys29, responsible for amyloid aggregation [70]. In this respect, HT seems to possess a dual protective role as able to inhibit both toxic amyloid formation and protein glycation reaction.

**Figure 9:**
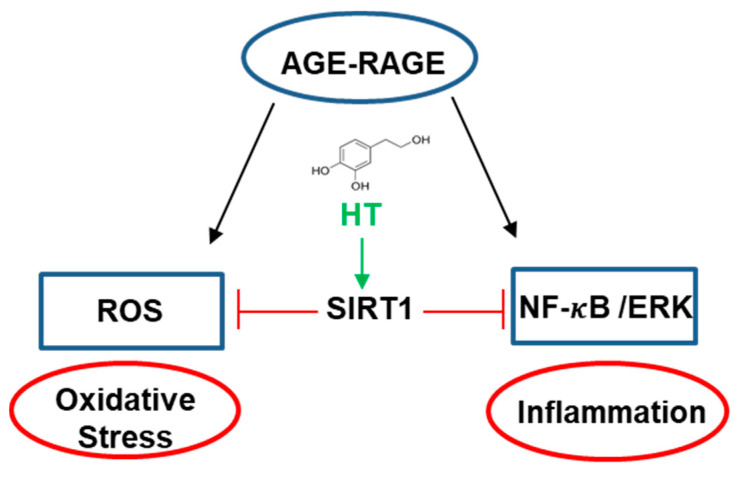
Working hypothesis on the protection effect of HT in AGE cytotoxicity. HT inhibits oxidative stress and inflammation response through the upregulation of SIRT1 expression.

Finally, as HT is known for its antioxidant and anti-inflammatory activity, we have tested the protective effect of HT on the toxicity induced by glycated species as this aspect was never explored before [54,55,56]. Interestingly, our results clearly show that HT exerts a protective effect on AGE-induced cytotoxicity even at micromolar concentrations. We have previously reported that glycated insulin strongly affects the cell viability, promoting a death pathway consisting in oxidative stress, apoptosis, and inflammatory response activation [65,69]. Our data show that, beside from its protective effect on cell mortality, HT is able to inhibit the AGE-induced ROS production when co-incubated with glycated insulin. The HT antioxidant effect on AGE-toxicity seems to be related to the ability of HT to specifically affect the SIRT1 level as the presence of HT upregulates SIRT1 expression thus keeping active the cellular antioxidant pathways. These observations are further supported by recent data indicating that HT is able to regulate SIRT1 expression in vascular adventitial fibroblasts also modulating the inflammatory response [81]. Moreover, our data show that HT significantly reduces the AGE-induced activation of NF-κB and ERK 1/2 MAP kinase, thus suggesting inhibition of inflammatory pathways. In this respect, as SIRT1 markedly protects cells from oxidative stress injury and also hinders inflammatory pathways by inhibition of NF-κB [73,74], its upregulation by HT could be responsible for the cellular protection. Although further studies are needed to better elucidate this correlation, we could hypothesize that the HT protective effect in AGE toxicity is exerted through a SIRT1-dependent pathway [50,82,83,84,85] (Figure 9).

## 5. Conclusions

The overall data identify HT as a potential inhibitor of glycation reaction as well as a promising molecule in protecting against the AGE-induced cellular dysfunctions, a key mechanism underlying the development and progression of neurodegenerative disorders. These findings indicate that the beneficial effects of olive oil polyphenols, including HT, observed in neurodegenerative diseases, may arise from multifunctional activities, thus suggesting a possible use of HT as nutraceutic in the prevention and/or treatment of AGE-related diseases, especially in consideration of its good safety profile and the ability to cross the blood brain barrier [85].

## Data Availability

Data is contained within the article.
